# Large-Beam Picosecond Interference Patterning of Metallic Substrates

**DOI:** 10.3390/ma13204676

**Published:** 2020-10-20

**Authors:** Petr Hauschwitz, Dominika Jochcová, Radhakrishnan Jagdheesh, Martin Cimrman, Jan Brajer, Danijela Rostohar, Tomáš Mocek, Jaromír Kopeček, Antonio Lucianetti, Martin Smrž

**Affiliations:** 1 HiLASE Centre, Institute of Physics, Czech Academy of Sciences, Za Radnici 828, 25241 Dolni Brezany, Czech Republic; dominika.jochcova@hilase.cz (D.J.); r.jagdheesh@hilase.cz (R.J.); martin.cimrman@hilase.cz (M.C.); jan.brajer@Hilase.cz (J.B.); danijela.rostohar@hilase.cz (D.R.); tomas.mocek@hilase.cz (T.M.); antonio.lucianetti@hilase.cz (A.L.); martin.smrz@hilase.cz (M.S.); 2 Faculty of Nuclear Sciences and Physical Engineering, Czech Technical University in Prague, Brehova 7, 115 19 Prague, Czech Republic; 3 Institute of Physics of the Czech Academy of Sciences, Na Slovance 2, 182 21 Prague, Czech Republic; kopecek@fzu.cz

**Keywords:** Perla laser, interference patterning, LIPSS, superhydrophobicity

## Abstract

In this paper, we introduce a method to efficiently use a high-energy pulsed 1.7 ps HiLASE Perla laser system for two beam interference patterning. The newly developed method of large-beam interference patterning permits the production of micro and sub-micron sized features on a treated surface with increased processing throughputs by enlarging the interference area. The limits for beam enlarging are explained and calculated for the used laser source. The formation of a variety of surface micro and nanostructures and their combinations are reported on stainless steel, invar, and tungsten with the maximum fabrication speed of 206 cm^2^/min. The wettability of selected hierarchical structures combining interference patterns with 2.6 µm periodicity and the nanoscale surface structures on top were analyzed showing superhydrophobic behavior with contact angles of 164°, 156°, and 150° in the case of stainless steel, invar, and tungsten, respectively.

## 1. Introduction

Fabrication of a functional surface with micro and nanostructures is a popular topic in science and industry. The surface functional performance often originates in hierarchical structures composed of micro and nanoscale features assembled into complex structures [1]. Their advantageous structure–property–performance relations have inspired extensive efforts for the study and development of methods for their effective replication. Among different type of structures, strategies for the fabrication of multi-scale structures have been the most extensively developed. Such structures can enhance or completely modify the original surface properties to achieve anti-icing [2], anti-corrosion [3], anti-bacteria [4], or superhydrophobic [5,6] properties.

Among a large variety of techniques for replicating naturally occurring microstructures, including chemical vapor deposition [7], chemical etching [8], sol-gel [9], plasma treatments [10], lithography [11], or electrodeposition [12], laser surface texturing provides a flexible, fast, and environmentally friendly approach for the high precision fabrication of desired micro and nanogeometries [13,14]. However, commonly used direct laser writing techniques, utilizing only one laser beam in a combination with a galvo scanner, may not satisfy the high industry demands for the treatment of large area in a short time. This is especially difficult to fulfil in the case of high-resolution micron scale structuring. Additionally, the increase in the average power of the ultrashort laser systems in recent years [15] were not reflected in the effectiveness of micro and nanoprocessing, as only a small percentage of the available laser power is used for processing to maintain high quality and avoid melting and thermal effects.

Direct laser interference patterning (DLIP) provides a solution to these issues by overlapping two or more coherent laser beams on the surface to directly imprint an interference pattern. If enough pulse energy is used, a large variety of materials can be processed at interference maxima positions, including metals [16,17,18], dielectrics [19,20], and even composite materials [21]. This allows the patterning of an area on the order of several tens of µm in diameter in a single irradiation step and with resolutions below the diffraction limit of the initial beam. The interference pattern can be controlled by the polarization, number of laser beams, and their incidence angle as well as by the wavelength, intensity, and phase difference. However, micron-sized pattern periodicity requires lenses with a short focal length to reach a high enough angle between overlapping beams. Consequently, potential throughputs are limited by the diameter of interference area, which is often on the order of a few tens of microns [16,21,22].

The choice of a laser system and its pulse duration plays an important role. Nanosecond systems are often used due to the less demanding DLIP setup precision and more flexibility in the setup design in general. The interference area can be larger compared to ultrashort pulses [23]. However, a lower temporal energetic density and the melting and thermal effects connected with nanosecond pulses limits the minimal achievable structure size and quality [16,24]. In addition, high beam quality and spatial coherence are also important parameters for high-quality DLIP structuring.

In line with that, this research presents a simple method that can increase the interference area diameter and preserve the high-resolution structures at the same time without any change in the optical setup. In particular, the high beam quality, ultrashort pulse duration below 2 ps, and high pulse energy of a HiLASE Perla laser in a combination with a novel technique of enlarging the interference area within the boundaries of the laser system parameters are novel aspects compared to the more commonly applied two beam configurations [16,21,24]. By utilizing this method, high throughput fabrication of micron and sub-micron sized features is demonstrated on several metallic materials, including stainless steel, commonly used in industrial contexts, such as medical implants, precision mechanics, marine applications, and food handling; tungsten, with applications in the production of hard materials and coatings; and invar, used in electronics and precision mechanical systems where a high degree of dimensional stability is required under a wide range of temperatures. 

## 2. Principles of Interference Patterning

In order to predict the energy distribution inside the interference area, as well as the effective overlapped beam size for interference, basic simulation was performed using the following equations.

Considering the wave approximation, the total complex amplitude of the electric field in a position $\vec{r}$ is given by a superposition of contributions of individual beams i.e., plane waves.

(1) $$I\left(\vec{r}\right)\propto {\left(\overset{N}{\underset{i=1}{\sum }}{\vec{E}}_{i}(\vec{r})\right)}^{2}$$

where $\vec{r}$ is the coordinate vector, *t* is the time, *i* is the index of interfering beams, *N* is the number of beams, and ${\vec{E}}_{i}$ is the electrical field of *i* beam, which can be expressed as follows:

(2) $${\vec{E}}_{i}={\vec{E}}_{0i}\;\cos\left({\vec{k}}_{i}\cdot \vec{r}-\omega t+\varphi_{i}\right)$$

where $\left|{\vec{E}}_{0i}\right|$ is the amplitude of *i* wave, $\left|{\vec{k}}_{i}\right|=2\pi /\lambda$ is the wave vector of the *i* wave, λ is the laser wavelength, ω is the frequency of radiation, and $\varphi_{i}$ is the phase of the *i* wave.

The total intensity in a position $\vec{r}$ is proportional to the squared module of the complex intensity vector

(3) $$I\left(\vec{r}\right)\approx {\left|\vec{E}\left(\vec{r}\right)\right|}^{2}.$$

In the case that all interfering beams have the same frequency, periodical intensity field is formed, and the equations can be simplified to [25]:

(4) $$I\left(\vec{r}\right)\propto \frac{1}{2}\sum_{i=1}^{N}{\left|{\vec{E}}_{0i}\right|}^{2}+\sum_{j<1}^{N}\sum_{i=1}^{N}{\vec{E}}_{0i}\cdot {\vec{E}}_{0j}\;\cos\;({\vec{k}}_{i}\cdot \vec{r}-{\vec{k}}_{j}\cdot \vec{r}+\varphi_{i}-\varphi_{j}).$$

As observed from Equation (4), the interference period depends on the incident angle between the beams and on the wavelength. Therefore, the lateral dimensions of the interference pattern (spatial period Λ) can be controlled by the angle θ between incident laser beams [26]. For two beams, the spatial period Λ can be calculated using Equation (5):

(5) $$\Lambda =\frac{1}{2}\frac{\lambda }{\sin\left(\theta /2\right)}.$$

Thus, structures with micro and sub-micrometer periodicity can be reached with common laser systems emitting at ~1 µm without any special objectives.

**Figure 1:**
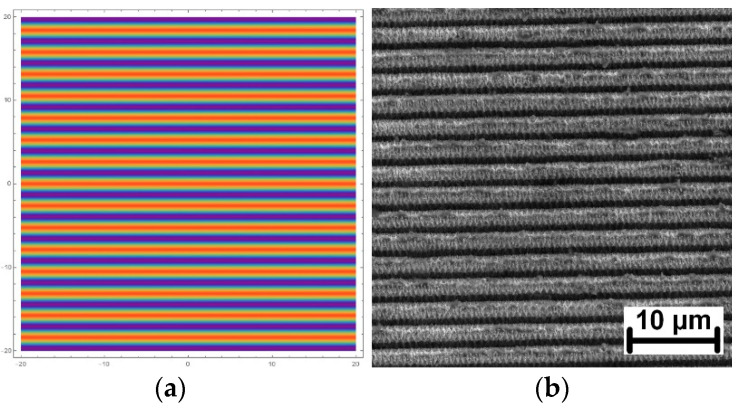
(**a**) Calculated pattern for two beam interference with a periodicity of 2.64 µm; (**b**) SEM image of the pattern fabricated on stainless steel with the same periodicity.

Good agreement between simulations (Equation (4)) and the pattern fabricated on a stainless-steel surface is demonstrated in Figure 1.

Higher throughputs can be reached with a larger beam diameter. However, the maximal reach of the interference area inside the overlapped beam region is limited by the pulse duration. For two beams overlapping at angle θ, the diameter of the interference area influenced by the pulse duration can be estimated by Equation (6) [23]:

(6) $$d\approx \frac{c\tau }{\sin\left(\theta /2\right)}$$

where *c* is the speed of light, and *τ* is the pulse duration.

Another important aspect of the pulse duration relates to the heat diffusion length $l_{H}$, which limits the minimal achievable structure size due to melting [16]:

(7) $$l_{H}=\sqrt{\frac{K\cdot \tau }{\rho \cdot c_{p}}}$$

where K is the thermal conductivity, ρ is the material density, and $c_{p}$ is the specific heat capacity. These material constants are shown in Table 1 for the materials used in this research.

Therefore, there is a tradeoff between the interference area and heat diffusion length. The shorter the pulse duration, the finer the structures that can be produced, but, at the same time, the smaller the area of interference. According to Equation (7), the heat diffusion length for tungsten is 2.6 µm for a 100 ns pulse duration and only 3 nm in the case 100 fs. The corresponding diameter of the interference area d is 153 m and 154 µm for 100 ns and 100 fs pulses (θ = 22.5°), respectively.

## 3. Materials and Methods 

**Figure 2:**
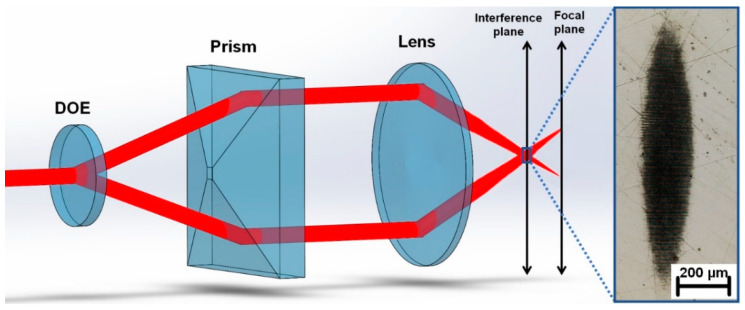
Illustration of the experimental setup with a marked shift between the interference and focal plane and with an inset of the elongated spot shape fabricated on a stainless-steel sample.

Different metallic materials, including stainless steel 316L, tungsten, and invar (FeNi36), provided by Goodfellow GmbH, were used for interference patterning. The sample thickness was 5 mm in the case of stainless steel and 0.2 mm in the case of invar and tungsten. A ytterbium-based diode pumped solid state laser system Perla from HiLASE emitting ultrashort pulses with a pulse duration of 1.7 ps and M^2^ of 1.2 at 1030 nm wavelength was used for DLIP processing. The incident laser beam was split into two beams by a diffractive optical element (DOE) with a separation angle of 3.97° (Holo/Or Ltd.). In the next step, the beams were parallelized by a prism and focused on a sample using a lens with a 60 mm focal length. The prism position was adjusted to reach the periodicity of 2.64 µm (θ = 22.5°). The illustration of the experimental setup is shown in Figure 2.

The pulse duration of 1.7 ps implies a large enough area for interference with diameter of 2.6 mm together with a heat diffusion length below 12 nm for all selected materials. Another advantage of pulse duration below ~10 ps is the possibility to produce laser induced periodic surface structures (LIPSS) on top of DLIP geometry and further enhance the functional properties of the structured surface.

To increase the throughput, the divergence of the initial laser beam was modified by telescope causing a shift between the interference plane and the focal plane, thus, elongating the interference area and reshaping it into an elliptical area due to the high angle θ (Figure 2). The divergence was tuned with the goal of reaching a major ellipse diameter of ~1 mm, which is well below the calculated limit of 2.6 mm and allows the use of reasonable pulse energies. Pulse energy up to 3 mJ and a 1 kHz repetition rate were used for this research.

Immediately after patterning, the samples were stored in a high vacuum (10^−7^ Pa) for 4 h to promote favorable chemical changes and decrease the transition time to reach a superhydrophobic state. The surface morphology was investigated using a scanning electron microscope, Tescan FERA 3 at electron energy of 5 kV, and a laser scanning confocal microscope, Olympus OLS5000 (Olympus Corporation, Tokyo, Japan). The wettability was evaluated by means of static contact angle measurements of 8 µL sessile droplets using the optical contact angle measuring device OCA 15EC (Data Physics Instruments, Filderstadt, Germany).

## 4. Results and Discussion

**Figure 3:**
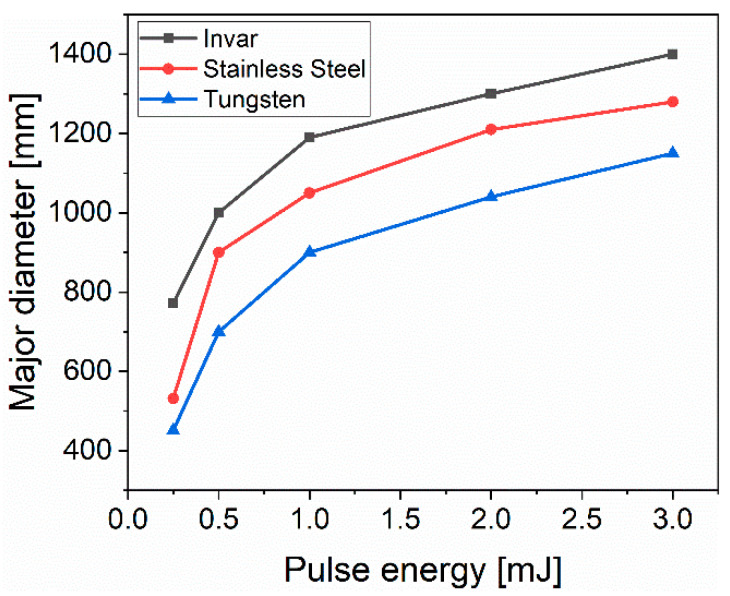
The major diameter of the elliptical interference area with a dependence on pulse energy.

Figure 3 presents the evolution of the major ellipse diameter with the applied pulse energy for 1000 consecutive laser pulses. As can be observed, a higher amount of accumulated energy resulted in a larger diameter for all materials and, thus, potentially improves the fabrication speed. Variations of the major ellipse diameter for the same applied laser parameters but different materials were caused by the different ablation thresholds of each material. The largest major diameter of 1.40 mm was achieved for invar irradiated with 3 mJ (2.44 J/cm^2^) and 1000 consecutive laser pulses. With the same laser parameters, the major diameters of stainless steel and tungsten were 1.28 mm and 1.15 mm, respectively.

**Figure 4:**
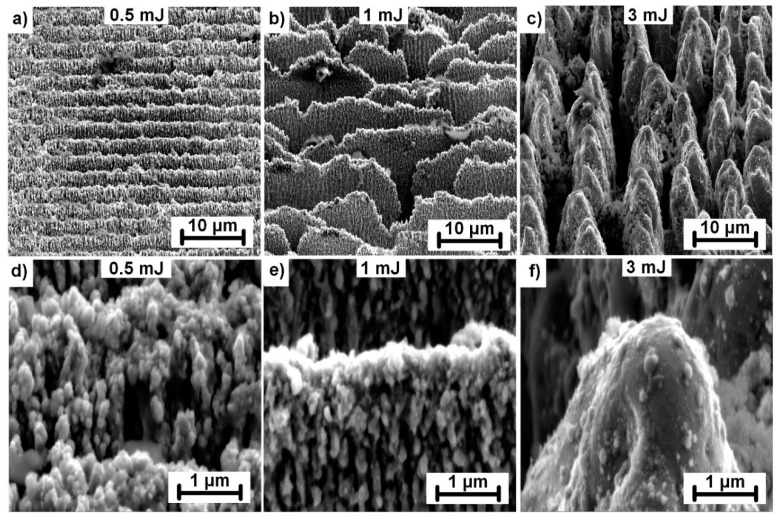
Structure evolution on a stainless-steel surface with increased pulse energy and 1000 consecutive laser pulses. Detail images with higher magnification are shown in the second row with the corresponding pulse energy. All images were captured with the sample tilted 55°.

However, the morphology of the produced structures also changed with the increase in pulse energy. Three different types of structures were identified at energy levels between 0.5 mJ (0.41 J/cm^2^) and 3 mJ (2.44 J/cm^2^) for samples irradiated with 1000 consecutive laser pulses, as depicted in Figure 4 for stainless steel.

For pulse energies below 1 mJ (0.81 J/cm^2^), a periodic pattern in the form of lines separated by 2.64 µm were fabricated with a groove depth of approximately 1 µm (Figure 4a). In addition, the whole surface was covered with nanoscale protrusions originating from melting and melt ejections on the top surface layers (Figure 4d), thus, generating dual-scale hierarchical structures. At pulse energies close to 1 mJ, the grooves began to break (Figure 4b). The amount of nanoscale protrusions decreased. The highest applied pulse energy of 3 mJ resulted in a formation of ~4 µm wide micropillars with a significantly reduced number of nanoscale protrusions. The appearance of micropillars can be explained by altering the energy distribution of incoming laser pulse on broken groove structures. Broken grooves may act as precursor sites, scattering the incoming light, thus, activating the preferential valley ablation between precursor sites. In the following step, conical-shaped micropillars were formed and grew with incoming laser pulses due to melt flow from the valleys towards the micropillar peaks. The melt flow is driven by thermal gradients caused by inhomogeneous energy distribution induced by the surface geometry. This can be evidenced by smooth micropillar walls with a minimum amount of nanostructures indicating the melt flow on the surface (Figure 4f). Similar structures were observed on nickel irradiated by a femtosecond laser system [27].

**Figure 5:**
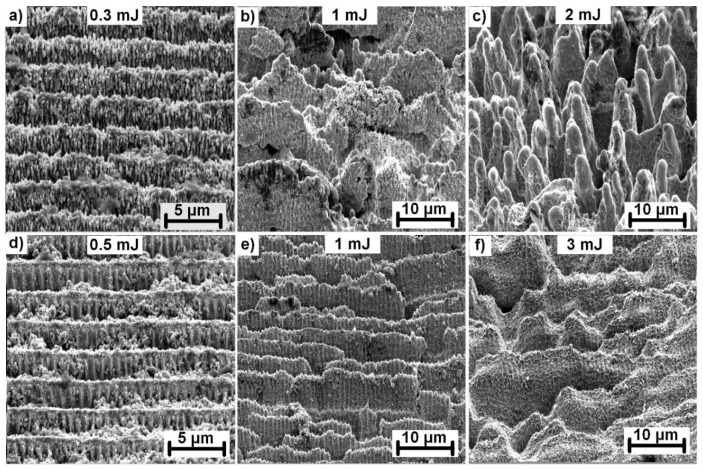
Structure evolution in Invar (**a**–**c**) and tungsten (**d**–**f**) with increasing pulse energy. The surface was irradiated with 1000 consecutive laser pulses. All images were captured with the sample tilted 55°.

As presented in Figure 5, it was possible to manufacture similar surface structures on invar (Figure 5a–c) and tungsten (Figure 5d–f) by slightly adjusting the pulse energy in the rage of 0.3 mJ (0.24 J/cm^2^) to 2 mJ (1.62 J/cm^2^) for invar and 0.5 mJ (0.41 J/cm^2^) to 3 mJ (2.44 J/cm^2^) for tungsten. Comparing the structure evolution, the morphology of invar structures followed the same trend as stainless-steel structures. The exception is the distinctive pulse energy levels, which were lower for invar. Contrarily, the amount of nanoscale protrusions covering the top surface was significantly lower in the case of tungsten. Conical shaped micropillar structures were not achieved on tungsten. With increasing pulse energy, the periodical structures start to break, however, further increases lead to melting and structure collapse instead of the formation of micropillars. In addition, the surface is covered with splashed and resolidified melt material in the case of the highest applied pulse energy.

**Figure 6:**
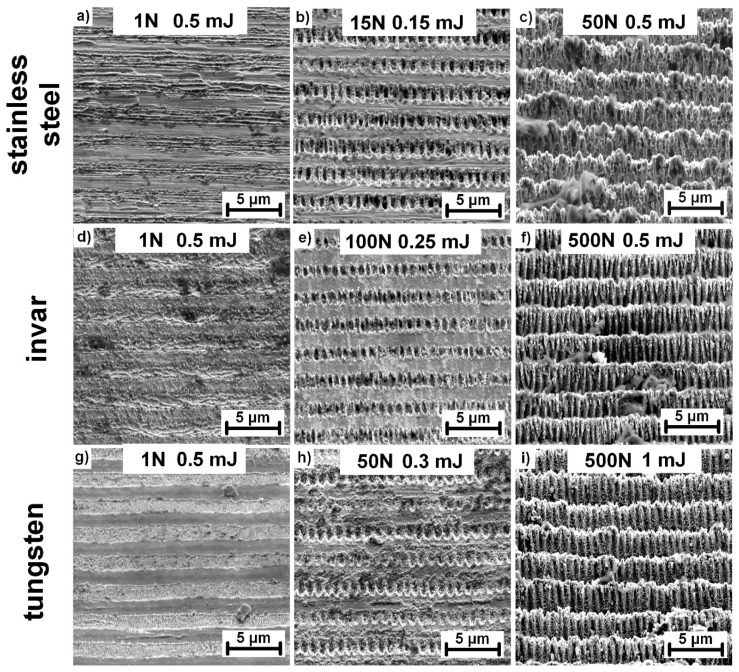
Different surface morphologies achieved by variations in the pulse energy and number of pulses (N) on invar (**a**–**c**), stainless steel (**d**–**f**), and tungsten (**g**–**i**). All images were captured with the sample tilted 55°.

Based on these results, a detailed analysis of the structure morphology was carried out by changing the number of pulses as well as the pulse energy. Consequently, new surface morphologies were observed as depicted in Figure 6.

**Figure 7:**
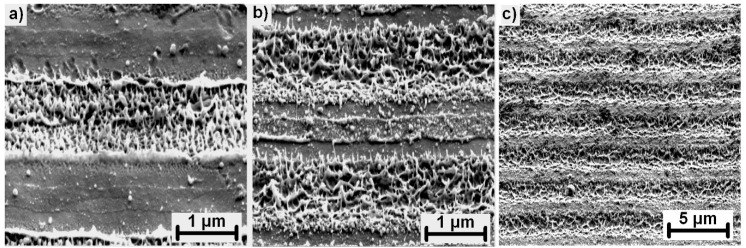
High magnification images of a tungsten surface irradiated with 1 mJ (0.81 J/cm^2^) and a single pulse (**a**), 5 pulses (**b**), and 10 pulses (**c**). All images are captured with the sample tilted 55°.

For a single pulse treatment, periodically arranged lines with lateral spacing of ~400 nm were observed with the orientation parallel to the polarization of the laser beam. Considering the characteristic size and orientation of these structures and parameters of the laser source (1030 nm, 1.7 ps), these structures can be identified as high spatial frequency LIPSS (HSFL) [28]. They are produced only at the interference maxima positions, thus, with an adjustable separation distance following the interference period, HSFL were observed in the case of invar and stainless steel, as depicted in Figure 6a,d. HSFL were more pronounced in the case of invar for pulse energies in a range of 0.25 mJ (0.2 J/cm^2^) to 0.5 mJ (0.41 J/cm^2^) with a lower amount of melting. In the case of stainless steel, substantial melting at the interference maxima disrupted the straight lines. Contrarily, melting and melt ejections were the main mechanisms observed for the single pulse treatment of tungsten, which prevented the formation of HSFL, however, showing promise for melt induced nanostructure fabrication. Therefore, a more detailed look at the melt evolution is presented in Figure 7.

A higher number of applied pulses resulted in another set of periodical arrays formed at the interference maxima positions. These can be referred to as low spatial frequency LIPSS (LSFL) due to the perpendicular orientation of the laser beam polarization and periodicity of ~870 nm, which is comparable with the used wavelength [28]. LSFL can be observed for all treated materials with 50 and more consecutive pulses at pulse energies of 0.15 mJ (0.12 J/cm^2^) for invar and 0.25 mJ (0.2 J/cm^2^) and 0.3 mJ (0.24 J/cm^2^) for stainless steel and tungsten, respectively.

As observed in Figure 7, a detailed look at the surface morphology on tungsten during the first few pulses revealed a different structure evolution. HSFL were not produced during the single pulse irradiation. The material appeared to be melted with random nanostructures originating from rapid melting and melt ejections toward the laser source, followed by quick resolidification forming randomly organized nanopillars with dimensions below 100 nm (Figure 7a). The following pulses caused remelting and merging of these nanostructures (Figure 7b). With the increased number of pulses, these structures were agglomerated and flattened until they covered the whole surface as shown in Figure 7c or Figure 5f.

To demonstrate the potential as a functional surface, the wettability of selected surfaces was tested. Tests were performed on all materials covered by similar structures composed of interference pattern with 2.6 µm periodicity covered with nanoscale protrusions on top with the same parameters as shown in Figure 4a for stainless steel, in Figure 5a for invar, and in Figure 5d for tungsten. All processed samples were placed for 4 hours into a vacuum chamber with the high vacuum of (10^−7^ Pa) to promote favorable chemical changes that may significantly decrease the transition time required to reach a superhydrophobic state. More details about the vacuum processing technique can be found elsewhere [29]. After that, the wettability was measured using the sessile drop technique with a droplet volume of 8 µL. 

**Figure 8:**
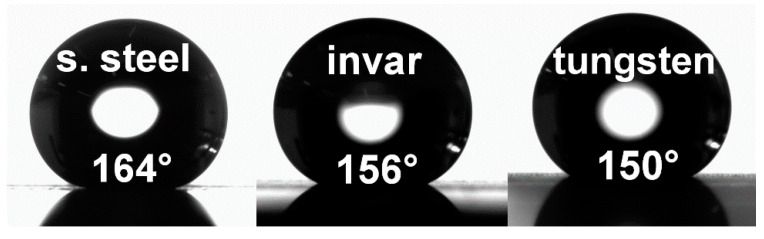
The wettability measurements of laser processed samples after vacuum treatment.

All samples were found to be superhydrophobic with contact angles of 164°, 156°, and 150° for stainless steel, invar, and tungsten, respectively, as shown in Figure 8. Therefore, hierarchical structures combining micro and nanoscale surface features exhibited excellent water resistance compared to untreated surfaces with a contact angle of 90°. This phenomenon can be explained by the Cassie–Baxter model [30] describing a rough surface that is able to trap air between surface features when a droplet is deposited on the surface. As a result, air trapped in between surface features prevents the water droplet from penetrating inside the valleys. Consequently, the water droplet is suspended only on the top of the microscale features, keeping the valleys dry and, thus, enhancing the water repellency.

The fabrication speed of introduced large-beam interference patterning technique reached 0.34 cm^2^/min (1000 consecutive pulses) and 206 cm^2^/min (single pulse). To reach a homogeneous pattern distribution, 30% pulse-to-pulse overlap (the spot center distance dived by the spot diameter) was experimentally found to be the best for seamless stitching over larger areas. Considering the 1 kHz repetition rate of the used laser system, these throughputs can be scaled up in orders of magnitude together with the repetition rate. For example, HiLASE Perla C [15] operated at 100 kHz and a 3 mJ pulse energy may theoretically speed up those fabrication speeds toward 34 cm^2^/min and 2.06 m^2^/min. The important benefit of this large-beam interference approach is the scalability. With enough laser power available, the area of interference can be further increased to reach higher throughputs. Thus, this technique shows promise for future applications of rapid large-scale surface functionalization.

## 5. Conclusions

A technique for increasing the throughput and efficiency of DLIP was introduced. By changing the beam divergence, the elliptical interference area with a major diameter above 1 mm and interference maxima in a form of lines oriented perpendicular to the major diameter was demonstrated. In addition, we used the basic principles of interference and performed calculations to determine the limits of the interference area and its dependence on laser parameters. A simple change in the laser parameters, including the pulse energy and number of pulses led to the fabrication of a variety of micro and nanostructures and their combinations produced in a single step on several metallic substrates. 

Pulse energies around 0.5 mJ with 1000 consecutive laser pulses resulted in a periodical groove structure covered by nanoscale protrusions, began to break at around 1 mJ of applied pulse energy and formed conical micropillar structures when the pulse energy increased above this level. Further examination revealed the parameters for the fabrication of HSFL, LSFL, and melt-induced nanostructures formed at the interference maxima positions, with an adjustable separation distance following the interference period. The fabrication speed of micro and nanostructured surfaces reached up to 206 cm^2^/min showing promises for the future rapid large-scale fabrication of functional surfaces by high-power laser systems. In addition, surfaces covered by DLIP-induced groove structures combined with nanoscale protrusions exhibited superhydrophobic properties after vacuum treatment with contact angles of 164°, 156°, and 150° in the case of stainless steel, invar, and tungsten, respectively.

## Figures and Tables

**Table 1:** Material constants provided by the supplier (Goodfellow GmbH) and heat diffusion lengths calculated for 100 ns and 100 fs pulse durations.

Material Constant	Stainless Steel	Invar	Tungsten
K $\left[{W\;m}^{-1}K^{-1}\right]$	16.3	13	173
ρ $\left[{\mathrm{Kg}\;m}^{3}\right]$	7960	8000	19300
$c_{p}$ [Kg K]	502	515	133
$l_{H\;\tau =100\;ns}$ [nm]	639	562	2596
$l_{H\;\tau =100\;fs}$ [nm]	1	1	3
